# Eco-Friendly Biopolymer Composite Sheet Derived from Water Hyacinth Reinforced with Cassava Chip: Optimal Conditions for Mixing, Blending, and Forming

**DOI:** 10.3390/polym17192709

**Published:** 2025-10-09

**Authors:** Praepilas Dujjanutat, Woravut Suwanrueng, Pakawadee Kaewkannetra

**Affiliations:** 1 Department of Biotechnology, Faculty of Technology, Khon Kaen University, Khon Kaen 40002, Thailand; praedujja@gmail.com; 2 Department of Agricultural Extension and Development, Faculty of Agriculture, Khon Kaen University, Khon Kaen 40002, Thailand; 3 Graduate School of Khon Kaen University, Khon Kaen University, Khon Kaen 40002, Thailand; woravut@kkumail.com; 4 Innovation Engineering Program, Faculty of Engineering, Khon Kaen University, Khon Kaen 40002, Thailand

**Keywords:** composite biomaterial sheet, water hyacinth, cassava chip, reinforcement

## Abstract

The persistence of the synthetic plastic waste problem makes it one of the most pressing environmental challenges. Sustainable material is an alternative approach to reduce petroleum plastics. In this research, our work aims to convert two biomaterials, water hyacinth (WH) and cassava chip (CC), into value-added biopolymer composite sheets (BCS). The raw materials of both WH and CC were prepared and characterized using physical and chemical treatments. Alkali treatments and chemical modifications were applied to remove lignin, protein, lipid, and other inhibiting components. After that, the two main raw materials of the WH and CC components were varied (100:0, 90:10, 80:20, 70:30, and 60:40, respectively) to investigate the optimal conditions for mixing, blending, and forming processes. Finally, mechanical properties (tensile strength), physical properties (surface morphology using a scanning electron microscope (SEM), crystalline structure by X-ray diffraction (XRD), and water solubility were also evaluated. The results obtained obviously revealed that the BCS reached an optimal ratio of 80:20 and exhibited outstanding properties. We were successful in exploring the potential use of a combination of two kinds of biopolymers under optimal conditions to produce an effective and environmentally friendly BCS in a manner that promotes a sustainable bio-circular economy and zero-waste concepts.

## 1. Introduction

Water hyacinth (WH), a free-floating aquatic plant of *Eichhornia crassipes* (Mart.) Solms, classified into the family Pontederiaceae, is a high and rapidly growing, widely spread, and strong plant. WH has been recognized by The International Union for Conservation of Nature as one of the most invasive weed plants in the world [1,2,3]. WH causes significant environmental and socioeconomic problems [4,5]; however, many researchers have tried to focus on converting excessive WH biomass into various value-added products, including biopolymers [2], bioenergy [3,6,7], compost and biofertilizer [8,9,10], and animal feeds [11]. Furthermore, WH is also applied in wastewater treatment plants for the adsorption of heavy metals such as arsenic and chromium [12,13,14]. Moreover, WH can be converted into diverse bioactive secondary metabolites such as phenolics, flavonoids, tannins, etc. [15].

Cassava, a resilient root crop of *Manihot esculenta* (L.) *Crantz*, was first introduced to Thailand in the early 1900s and has since evolved from a subsistence crop to a major economic driver. Currently, Thailand stands as the world’s third largest producer of it (after Nigeria and Brazil). The country’s cassava industry has developed from basic agricultural production into complex value-added processing, converting raw cassava into high-quality starch [16,17], animal feed pellets [11], ethanol [3], bio-composite cassava starch and edible starch-based films [18,19], bioplastics [17] and biodegradable packaging [20], pharmaceutical products, and cosmetic ingredients [21]. In Thailand, there are several cassava products, such as cassava starch, cassava pulp, and cassava chip (CC), that can be used for the production of biopolymers and bioplastics. Applications depend on price, composition, and objectives. Comparing them, cassava starch has the highest cost (1.16 USD/kg), then cassava pulp (0.47 USD/kg), and CC has the lowest price (0.26 USD/kg). Previous studies have paid attention to the use of cassava starch and WH for the production of biomaterials and biopolymers [20,21,22].

As investigations of sustainable alternatives to petroleum-based plastic products increase, WH and cassava starch are uniquely positioned to meet the growing demand for bio-based materials, including biopolymers and biodegradable packaging [23,24,25,26,27,28]. Our previous work successfully facilitated physicochemical conversions of WH; sugarcane bagasse and rice straw were applied to produce an innovative biopolymer of carboxymethylcellulose (CMC). Then, the CMC was blended with the biopolymer and plasticizer of cassava starch and glycerol, respectively, in order to form a bioplastic film [28]. However, the obtained film consisted of three main compositions. In order to reduce the cost of production, a low-grade and cheap raw material was used with the main purpose being its further application as biodegradable packaging. In this study, we are interested in using only two main components of CMC, derived from WH and CC, to produce the BCS. Therefore, the objectives of this study were as follows: The characterization of the two raw materials, WH and CC; secondly, to convert WH into cellulose and then into a biopolymer of CMC; and thirdly, to investigate the optimal conditions for the mixing, blending, and forming steps of the BCS. Finally, the BCS was evaluated in some physical, chemical, and mechanical properties.

## 2. Materials and Methods

### 2.1. Materials and Chemicals

The main raw materials used, such as samples of WH, were collected from natural water basins, while cassava samples in three forms and CMC as a control set were of commercial grade. All chemicals used throughout this study were analytical grade and purchased from Merch Thailand, Bangkok, Thailand, including sodium hydroxide (NaOH), hydrogen peroxide (H_2_O_2_), acetic acid (CH_3_COOH), isopropyl alcohol (C_3_H_8_O), sodium silicate (Na_2_SiO3), chloroacetic acid (C_2_H_3_O_2_Cl), methanol (CH_3_OH), and ethanol (C_2_H_5_OH). It should be noted that details of the chemicals used will be explained in the later topics regarding extraction and conversion.

### 2.2. Collection and Preparation of WH

Aquatic weed of WH was collected from natural water basins in Khon Kaen province, northeast of Thailand. Only root parts were cut and removed while the remaining parts were washed using tab water to remove soil and other contaminants. The procedure was explained by Ungprasoot et al. (2020) [28] as follows; WH samples were chopped into a small size and dried under sunlight for 24–48 h or using a hot air oven at 65 °C for 12 h. WH samples were ground by a grinder (capacity 2.5 kg/batch) and then sieved using screening (60 mesh). WH samples were obtained and kept in a desiccator prior to use in further steps.

### 2.3. Preparation of Cassava Chip (CC)

Samples of dried CC including cassava starch and cassava pulp were kindly received from a cassava factory located in Ubon Ratchathani province in the northeastern area of Thailand. CC samples were sieved using screening (no.5 mesh) to separate sand and contaminants. Then, they were ground using a grinder and screened again (no.60 mesh). CC samples were obtained and kept in a desiccator.

### 2.4. Characterizations of WH and CC

Both samples of WH and CC powder were characterized in their compositions. In addition, cassava starch and cassava pulp were also analyzed and compared to the CC. Some parameters and analytical techniques followed the standard methods explained by previous studies [15,28]; the details are shown in Table 1.

### 2.5. Extraction of Cellulose from WH Powder

WH powder samples were also extracted to recover only the desired cellulose. All chemicals used were of analytical grade. The serial procedures are described below.

#### 2.5.1. Lignin Removal

An alkaline treatment was used to remove lignin from WH powder samples. The modified procedure was explained by Sari et al. [29]. Firstly, 10 g WH powder and sodium hydroxide (NaOH) were used with a ratio of 1:50. The mixtures, in blue top bottles, were placed in a water bath under 80 °C for 4 h while the bottles were shaken every 30 min. Only a liquid phase was removed while a solid phase was eluted by distilled water until pH reached 7. Then, it was dried in a hot air oven at 60 °C for 24 h. A dried cellulose was obtained and was ground using a blender before bleaching reaction.

#### 2.5.2. Bleaching Step

For the decolorization of remaining lignin and pigments, a mixture of 10% hydrogen peroxide (H_2_O_2_) and 10% acetic acid (CH_3_COOH) was mixed using a ratio of 1:1 (*v*/*v*) and stirred for 24 h. The mixture was filtered using a cotton sheet. Only the solid form was eluted using distilled water until pH reached 7. It was dried in a hot air oven (50 °C, 24 h). Dried cellulose was obtained and was ground again using screening (40 mesh).

### 2.6. Conversion of Cellulose into Carboxymethylcellulose (CMC)

Cellulose powder was weighed at 5 g and then added into a mixture containing 20 mL isopropyl alcohol (C_3_H_8_O), 9 g chloroacetic acid (C_2_H_3_O_2_Cl) and 40 mL NaOH (concentration 40%) for 30 min. The solution was heated up at 60 °C for 3 h. After that, 50 mL methanol (CH_3_OH, concentration 70%) was added with an adjusted pH of 7 and soaked in 70% ethanol (C_2_H_5_OH) using acetic acid. It should be noted that this step was repeated five times. Solution of ethanol was removed using a Whatman filter. The paper filter was dried in hot air oven at 60 °C for 12 h and dried CMC powder was obtained.

### 2.7. Optimal Ratios of CMC:CC for Mixing, Blending and Forming

Mixing ratios for preparing CMC and CC powders were varied in five levels (100:0, 90:10, 80:20, 70:30 and 60:40). Two components (a total weight of 5 g) were mixed in a beaker. Then, 25 mL distilled water was added. The mixture was stirred on a magnetic stirrer and heated at 80 °C for 10 min. It was poured onto glass plates (Petri dish) lined with a waxed paper. Then, it was placed in a hot air oven under 50 °C for 24 h until the BCS were completely dried and taken from the plates.

### 2.8. Characterizations of Composite Biopolymer Sheet

#### 2.8.1. Scanning Electron Microscope (SEM) Analysis

Composite biopolymer sheets in various ratios of CMC:CC (100:0, 90:10, 80:20, 70:30 and 60:40) were characterized in overall surface, morphology and microstructure using the SEM technique, (Thermo-Fisher Scientific, Bangkok, Thailand, Model: Quattro-S) operated using technical parameters such as an accelerating voltage or electron high tension (EHT) of 20 kV and I probe at 221 pA with a beam current of 250 μm. The fracture surface of the test sheets was cut into small rectangular pieces of approximately 5–10 mm × 5–10 mm. In addition, the working distance (WD) was set at 19 mm while a magnification range of 500× was applied for all samples in each ratio.

#### 2.8.2. X-Ray Diffraction (XRD)

An excellent technique of XRD was applied to analyze the cellulose structure, crystallinity, and purity. Fine WH powder (after 200 mesh screening or approximately 0.074 mm) was prepared, the XRD instrument (Examinart, Tucson, AZ, USA. Model: Fenix) was set for analysis and data collection (scan range of 5–90° (2θ), step size 0.02° and total scan time of 2–3 h). Duplication scans were run for reproducibility.

#### 2.8.3. Moisture Content

The moisture content of the BCS samples was calculated based on the weight loss in sheets after drying. Firstly, aluminum cups were dried in a hot air oven under 85 °C until a constant dry weight was reached. They were kept in a desiccator prior to use. The empty cups and composite biopolymer sheets (0.5 ± 0.02 g) were weighed (*A*_0_). Then, they were dried in a hot air oven under 105 °C for 3 h and kept in a desiccator. After that, they were weighed and recorded as (*A*_1_). Moisture content was calculated in Equation (1) as follows:

(1) $$\mathrm{Moisture}\;\mathrm{content}\;(\%)\;=\frac{A_{0}-A_{1}}{A_{0}}\times 100$$

#### 2.8.4. Water Solubility

Samples were determined by a method described by Ungprasoot et al. (2020) [28]. They were cut into small rectangular pieces of about 50 × 50 mm^2^. It should be noted that each experiment was measured in triplication. The sheets were dried at 65 °C for 24 h. All sheets were cooled down in a desiccator. The initial sheets were recorded (*W*_0_) and then immersed in 100 mL distilled water. Beakers were covered with parafilm and left at room temperature for 24 h. Water was filtered using Whatman No.1 paper and dried in a hot air oven at 80 °C until constant weight was reached. All sheets were left until cooled down and weighed again (*W*_1_). The water solubility was calculated using Equation (2).

(2) $$\mathrm{Water}\;\mathrm{solubility}\;(\%)=\frac{W_{0}-W_{1}}{W_{0}}\times 100$$

#### 2.8.5. Oxygen Transmission Rate (OTR)

All Composite biopolymer sheets in various ratios were considered in oxygen permeability rate. The protocol was set by Thai Packaging Centre (TPC), Thailand Institute of Scientific and Technological Research (TISTR), Bangkok, Thailand. The standard method of ASTM D3985-05, OTR through bioplastic sheet uses a coulometric sensor using oxygen permeability analyzer (Illinois Instruments Inc., Johnsburg, IL, USA, Model: Systech lllinois 8000). In brief, sheet samples were placed between two chambers while oxygen and nitrogen as carrier gases from upper and low chambers, respectively, were transmitted, mixed and passed to sensor. The condition for testing was measured at 23 °C with a relative humidity of 0% and for 24 h.

#### 2.8.6. Tensile Strength Testing

Composite biopolymer sheets in different ratios were measured in tensile strength using a universal testing machine (UTM) (Stable Micro systems, Surrey, UK). Sheet samples in each ratio were cut in a dimension of 3 × 5 cm^2^. The machine was operated using a test speed of 1 mm/s, post-test speed of 10 mm/s and for a distance of 20 mm. Force and elongation at break were recorded. Ultimate tensile strength (UTS) of peak stress was obtained. It should be noted that each ratio of sheets was tested in duplication.

## 3. Results and Discussion

### 3.1. Raw Materials of Water Hyacinth (WH) and Cassava Chip (CC)

**Figure 1:**
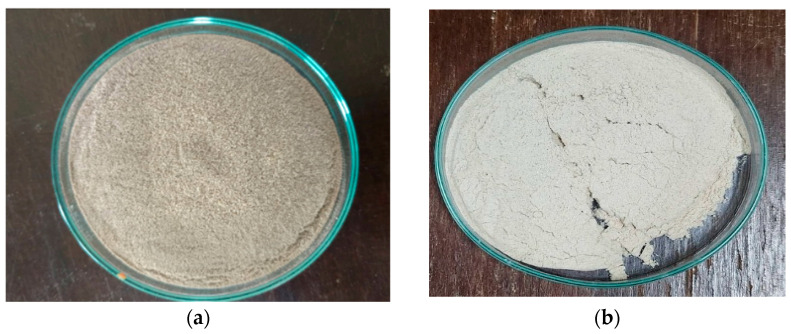
Raw materials after pretreatments; (**a**) WH powder, (**b**) CC powder.

After preparation, both raw materials of WH and CC were obtained in fine powder (approximately 0.3 mm). However, WH powder showed a yellow-brown due to lignin content in the powder while CC powder showed a cream color (See Figure 1a,b).

### 3.2. Characterizations of WH and CC

Both of WH and CC were characterized in their compositions as shown in Table 2.

Composition analysis of the WC and several forms of cassava was shown in Table 2. Cellulose, crude fiber and crude protein contents were found in maximum contents (35–40%, 17.67% and 6.9%, respectively) while crude lipid and lignin were found in quite small amounts because the root of the WH was cut off. For other compositions such as moisture content, total ash and non-fiber carbohydrates were also found. All of these can be used in selecting raw materials suitable for research or industrial applications, for example, the production of cellulose from the WH fibers. However, the WH has high protein and fat contents which could be affected on the properties of the fibers obtained. This information allows the selection of appropriate chemical reagents for further cellulose extraction. Meanwhile, the CC composition was shown in column 3. It was revealed that moisture content was found in the range of 10–11% which was a suitable value for storage. Starch contains in very high contents at about 76–78%, indicating that cassava is the main source of carbohydrates. Protein and fat contents were found at only 2–3% and 0.5–0.8%, respectively. In addition, dietary fiber in a range of 2–4% and insoluble ash of 2.6–3% were detected, which reflected on the minerals remaining after burning. In addition, cassava starch and cassava pulp were also analyzed and shown in columns 4 and 5, respectively. Only starch content was focused on due to its function as a plasticizer. The highest content was found in cassava starch while in the case of cassava pulp, half the amount was found. Compared to the CC, starch content was found in similar contents as cassava starch. Regarding the CC cost, it was cheaper than the cassava starch by about 2–3 times. It could be confirmed that the CC has a potential use to replace cassava starch to achieve our hypothesis in this current study of using a low-cost and low-grade raw material that can be blended with the CMC to produce the BCS containing only two main components.

Although the CC powder contains a high starch content, it is quite low in protein and fat content. Moreover, its acid-insoluble ash content may reflect impurities that must be controlled in the production process [28,29,30,31]. In addition, it should be noted that both WC and all forms of cassava were determined in duplication for reproducibility.

### 3.3. Characterizations of Cellulose Extract and Its Yield

**Figure 2:**
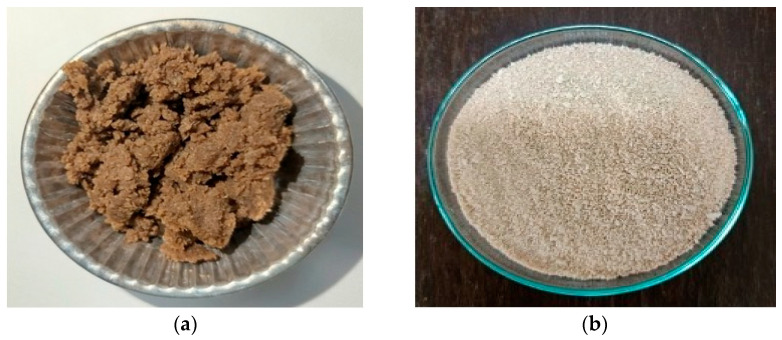
Cellulose extracted from WH (**a**) before bleaching and (**b**) after bleaching.

After bleaching, cellulose was extracted from WH. It was found that the cellulose color changed from brown to light-brown. Its external appearance was fluffy, lightweight and did not clump together, in contrast to its appearance before bleaching as shown in Figure 2a,b. Meanwhile, the cellulose yield was increased and obtained in a range of 45–50% (determination in duplication).

### 3.4. Characterizations of Carboxymethylcellulose (CMC) Extract and Its Yield

**Figure 3:**
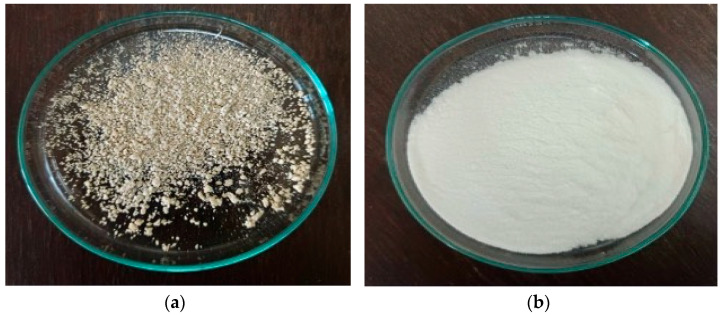
Cellulose conversion into (**a**) CMC from WH and (**b**) commercial-grade CMC.

After the conversion of cellulose, the CMC was obtained and compared to commercial-grade CMC. It was found that cellulose WH was converted by replacing a hydroxyl (-OH) group with a carboxymethyl group (-CH_2_COOH) for improving properties of water solubility and gel information, as shown in Table 3 and Figure 3, for physical appearance. Meanwhile, the CMC yield obtained was approximately 74.73–76% (determination in triplication). The results obtained were similar to a previous study by Ungprasoot et al. (2020) [28] who also studied the conversion of WH into the biopolymer of CMC and yielded at about 73–75%.

### 3.5. Composite Biopolymer Sheets

**Figure 4:**
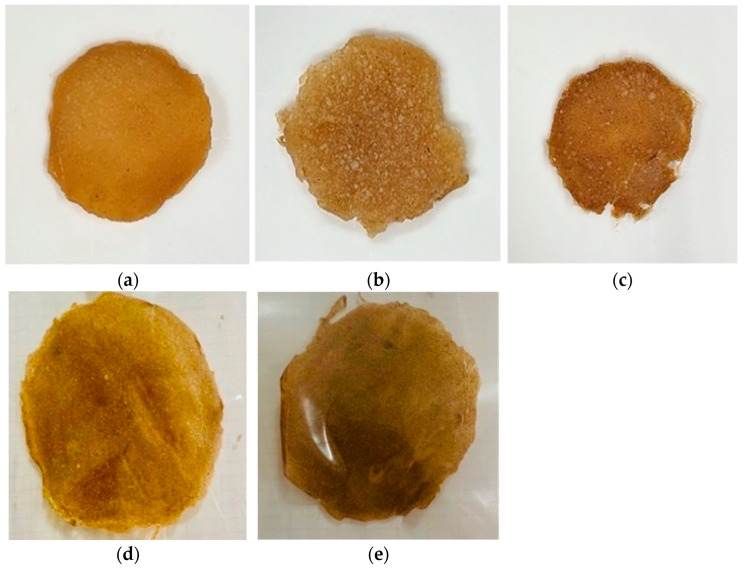
A set of composite biopolymer sheets in variations of CMC:CC, (**a**) 100:0 as a control set (**b**) 90:10 (**c**) 80:20 (**d**) 70:30 (**e**) 60:40.

Steps of mixing, blending, and forming of the BCS in variations of CMC:CC (100:0, 90:10, 80:20, 70:30, and 60:40) were performed as shown in Figure 4. It seemed that a ratio of 100% CMC showed good external surface area. However, the sheet with a ratio of CMC:CC (80:20) achieved the optimal condition after it was evaluated in other properties and good results were obtained, for example, moisture content and water solubility, the sheet showed a good moisture barrier property and its mechanical property of tensile strength still remained acceptable. It was summarized that as the amount of CC increased, the texture structure became rougher. The structural sheets obviously became a more complex agglomeration; this is due to the increase in the fiber content of cassava. The results obtained were in agreement with previous studies [17,18,24,32].

### 3.6. Scanning Electron Microscope (SEM) Technique

**Figure 5:**
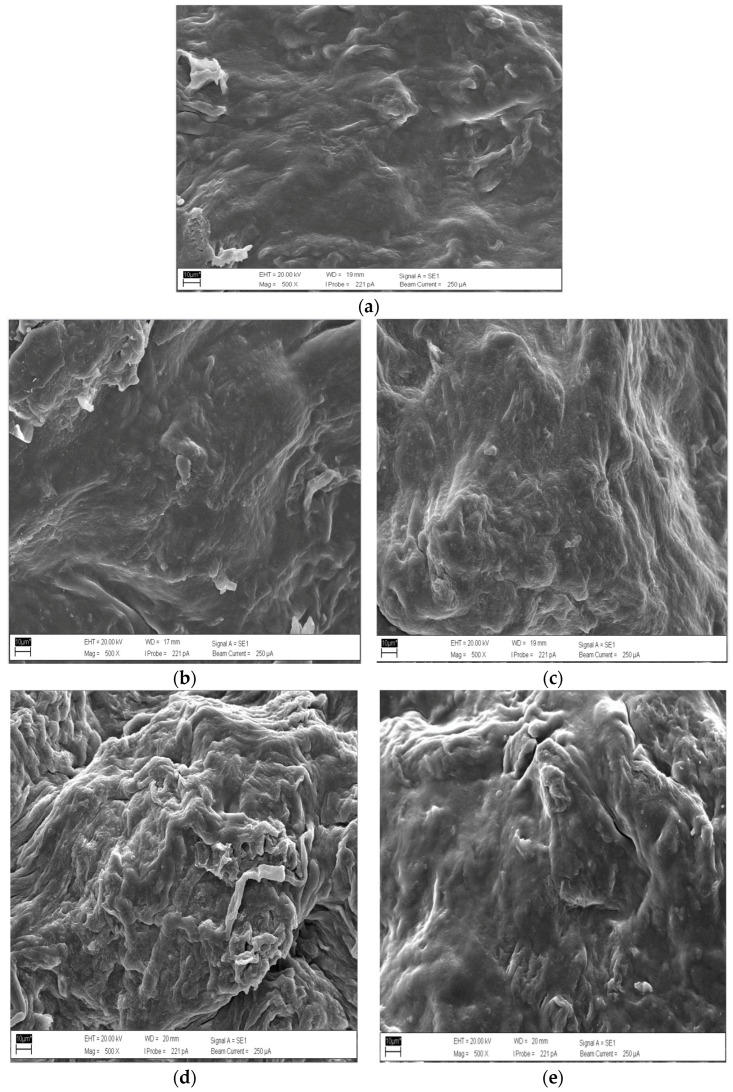
A set (**a**–**e**) of SEM images in different ratios of CMC:CC (100:0, 90:10, 80:20, 70:30, 60:40), respectively. (500× magnification).

In Figure 5, SEM images of the BCS revealed distinct morphological changes across different CMC:CC ratios at 100:0, 90:10, 80:20, 70:30 and 60:40, respectively. Typically, some key factors to evaluate SEM images include surface morphology and interface quality. In the case of pure CMC (100%) served as a control set (100:0), the sheet exhibited a relatively smooth, homogeneous surface and uniformity with minimal porosity, indicating forming properties. Moreover, there were no obvious agglomerations or structural differences. Meanwhile, at the ratio of 90:10, the surface was similar to the previous ratio (100:0) with slightly increased roughness. For the ratio of 80:20, the sheet showed wrinkles in the surface area. However, fibrous structure and clumps were found in the ratio of 70:30. The surface area was very rough, scaly and lumpy. Lastly, a combination of fibers and a non-homogeneous structure was seen in the ratio of 60:40. The surface exhibited highly rough and clearly lumpy areas. It could be summarized that as ratios increased (90:10 to 60:40), all BCS surfaces showed progressively rougher and more heterogeneous surfaces. At 10–20% CC additions, small particles were well dispersed within the CMC matrix, showing good compatibility between components. However, at higher ratios (30–40%), the CC particle embedded in the CMC appeared to have agglomerated and become embedded in the continuous CMC phase. Consideringly, only the SEM results clearly showed that a ratio of 80:20 represented the morphologically balanced surface. However, other properties including crystalline structure, moisture equilibrium, mechanical property, and solubility should be considered in further steps before achieving the optimal ratio for producing the BCS.

### 3.7. X-Ray Diffraction (XRD) Technique

**Figure 6:**
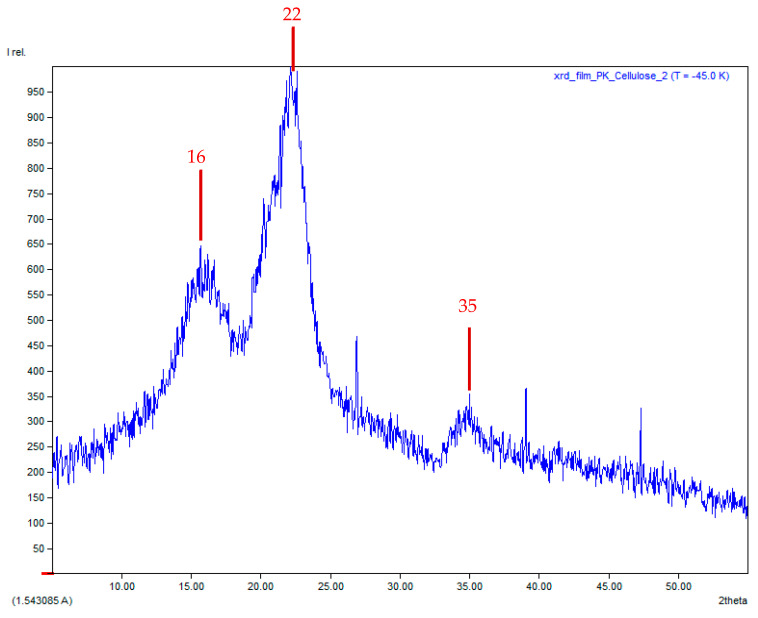
Characteristic peaks of cellulose extracted from water hyacinth (WH).

XRD is a crucial analysis for characterizing the BCS. XRD patterns indicate changes in crystallinity, identifying amorphous region formation and polymer chain rearrangement during blending and forming. Figure 6 shows the XRD technique, a well-established method for cellulose characterization. It was found that cellulose had a semi-crystalline structure and characteristic peaks showed around 16°, 22° and 35°. The XRD pattern obtained appeared corrected for the cellulose extracted from WH. Peak intensity pattern at 16° and 22° were of medium intensity and the strongest peak, respectively, and corresponded to native cellulose I that was the most abundant, occurring in a specific crystal structure, and usually found in the cell walls of plants [33]. Although the peak showed at 35° was weaker, but distinct, it was corrected and also classified as cellulose I.

**Figure 7:**
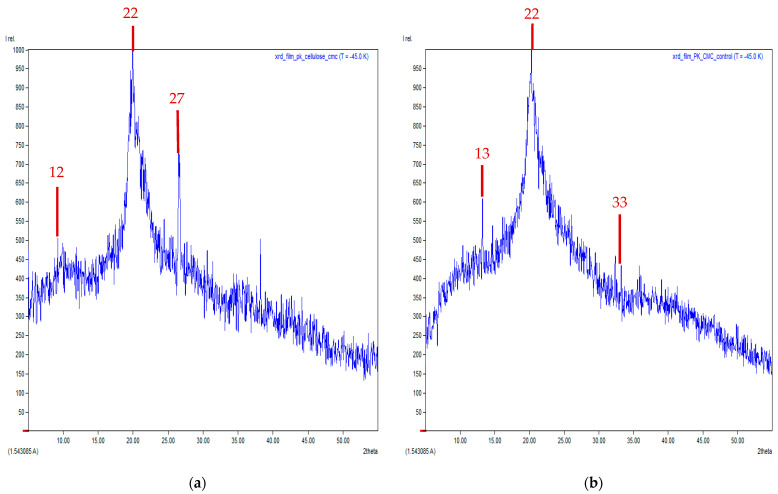
Characteristic peaks (**a**) commercial-grade CMC from (**b**) water hyacinth (WH).

Meanwhile, characteristic peaks of commercial-grade CMC were observed at 2θ, 12°, 22°, and 27° and compared to CMC converted from WH that was found at 2θ, 13°, 22°, and 33° (See Figure 7a,b). For peak positions of commercial-grade CMC, an expected peak typically centered at around 21–22°. This is in agreement with the results obtained only at 22° and while other peaks found were at 12° and 27°, it might not be characteristic and unusual for pure CMC. For the case of CMC extracted from WH, the peaks showed were similar to commercial-grade CMC and the main peak was observed at 22°. However, the peak at 13° was an unusual peak (not typical for cellulose or CMC) and the peak at 33° was close to cellulose but slightly shifted. It might be summarized that incomplete conversion of cellulose to CMC was observed and still retained a significant crystalline cellulose structure.

### 3.8. Moisture Content and Water Solubility

**Figure 8:**
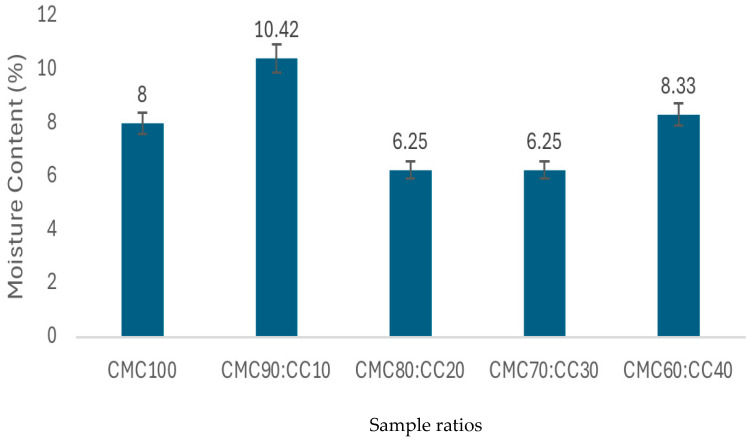
Moisture content of the BCS under variations in CMC:CC ratios (100:0, 90:10, 80:20, 70:30 and 60:40) (in duplication for reproducibility).

**Figure 9:**
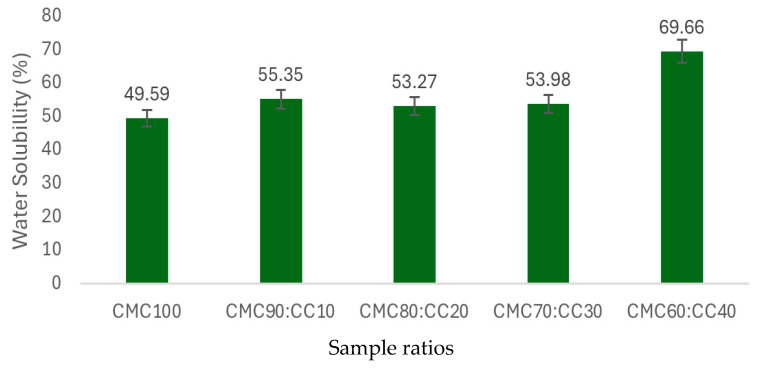
Water solubility of the BCS under variations in CMC:CC ratios (100:0, 90:10, 80:20, 70:30 and 60:40) (in duplication for reproducibility).

Moisture content (Figure 8) and water solubility (Figure 9) can be seen as important parameters for the BCS performance. Elevated moisture levels significantly compromise mechanical property by reducing tensile strength and stability. Meanwhile, water solubility directly limits application scope, particularly in humid environments. Analysis of moisture content and water solubility of the BCS in different ratios of CMC:CC are shown in Figure 8 and Figure 9. It was found that ratios of 80:20 and 70:30 reached the lowest moisture content while the highest moisture content was found at the ratio of 90:10. Ratios of CMC:CC at 100:0 and 60:40 reached similar levels of water solubility due to the maximum CMC and starch contained in CC, respectively. The results obtained were in agreement with previous studies mentioning that the hydrophilic nature of both CMC and CC resulted in high moisture absorption, leading to instability and reduced mechanical properties. This becomes a limitation that restricts outdoor applications and requires careful packaging and storage conditions [32,33,34,35]. Thus, the ratio of 80:20 was chosen due to a better moisture barrier property and stronger intermolecular interaction between CMC and CC.

### 3.9. Tensile Strength

**Figure 10:**
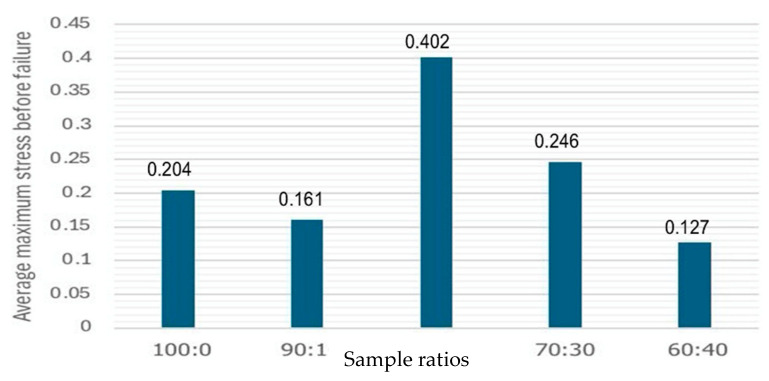
Average maximum stress as a function of ratios of CMC:CC in the BCS under variations in CMC:CC ratios (100:0, 90:10, 80:20, 70:30 and 60:40).

Importantly, the tensile strength often determines, as it directly impacts, the performance and applicability of BCS. Figure 10 showed the maximum stress (peak stress) or tensile strength in the BCS under variations in CMC:CC ratios (100:0, 90:10, 80:20, 70:30 and 60:40); the maximum stress level was found at 0.402 MPa. However, as ratios of CC increased, the stress was decreased and obtained at 0.127 MPa when the ratio of CMC:CC was 60:40. The results obtained were similar to a previous study by Anggraini et al. (2018) [36] who studied the mechanical properties of cassava-starch-based bio-composite reinforced with WH fiber. The expected values for biopolymer sheets of pure CMC and blended CMC:CC sheets were in a range of 0.5–2.0 MPa and 0.2–1.5 MPa, respectively [26,37]. While the optimal ratio of 80:20 obtained in the study was reached with a value of 0.042 MPa, it was within a reasonable range and agreed with previous studies [23,38]. In addition, it corresponded to the water solubility result. The BCS improved tensile strength and reduced water sensitivity.

## 4. Conclusions

Undoubtedly, the current study presents a novel approach to sustainable packaging by developing carboxymethyl cellulose (CMC) blends from two abundant, low-cost raw materials of water hyacinth (WH) and cassava chip (CC). The innovation lies in the strategic combination of only two materials where WH, typically considered as an invasive aquatic weed, is transformed into a valuable biopolymer component, while CC provides complementary mechanical properties. The optimal ratio for mixing, blending, and forming of the BCS between CMC and CC was achieved at 80:20. The BCS clearly displayed good properties including crystalline optimization, moisture balances between hydrophilic and hydrophobic interaction, mechanical reinforcement, and functional solubility.

## Figures and Tables

**Table 1:** Parameters and analytical techniques used to analyze WH, CC, cassava starch, and pulp.

Parameters	Analytical Technique
Cellulose	Acid Detergent Fiber (ADF)
Crude Fiber	Crude Fiber Analysis
Carbohydrate	Phenol-Sulfuric Acid Methods
Crude Protein	Lowry Method
Crude lipid	Acid Hydrolysis
Lignin	Acid Detergent Lignin (ADL)
Total ash	Muffle Furnace 550–600 °C

**Table 2:** Composition of water hyacinth (WC), cassava chip (CC), cassava starch, and cassava pulp.

Composition	Water Hyacinth (%)	Cassava Chip (%)	Cassava Starch (%)	Cassava Pulp (%)
Cellulose	35–40	4–5	0.1–0.5	10–28
Crude fiber	17.67	2–4	2–3	10–18
Starch	4.2	76–78	72–85	35–37
Crude protein	6.90	2–3	2–6	1.8–2.0
Crude lipid	1.18	0.5–0.8	0.05–0.45	0.4–0.5
Lignin	0.92	2–4	*	5–15
Total ash	33.92	2.6–3.0	1.5–3.0	3.5–3.7

* Not detected or found in very small amounts.

**Table 3:** Compositions of water hyacinth (WC) and cassava chip (CC).

Characteristics	CMC (from Water Hyacinth)	CMC (Commercial Grade)
Color	Yellow-brown	Light cream
External appearance	Fluffy and light weight	Light weight
Gel information	Moderate	Good
Solubility	Moderate	Good

## Data Availability

The original contributions presented in this study are included in the article. Further inquiries can be directed to the corresponding author.
